# Nurses' Emoluments, Hours, and Holidays

**Published:** 1918-10-12

**Authors:** 


### FORGING AHEAD.

### Nurses' Emoluments, Hours, and Holidays.

It is extremely gratifying to note that the progress of events in the nursing world is consonant with the march of our gallant armies to victory, and that clear indications of a brighter day are becoming visible. On Thursday of last week the Council of the College of Nursing formally entered on the solution of the economic problem, and as it now voices twelve thousand trained nurses, obviously any conclusions at which it may arrive will be invested with authority and force. I have always been convinced that no fundamental reforms can be effected in the nursing world until the workers' services are recognised by being fairly and adequately rewarded. The pressure of the war, and the demand for women workers in every field of labour, have made the neglected claims of the nurse more and more insistent, with the result that individual hospital boards have been endeavouring to find a way out. The authorities of the Charing Cross Hospital have suggested a conference of the chairmen of metropolitan hospitals "to discuss the whole question of emoluments, holidays, and hours of work" of nurses. The first essential step in the way of reform is the education of the public, and every step in this direction is all to the good. At the same time it is, I think, apparent that hospital chairmen could not constitute an adequately equipped tribunal to undertake an inquiry. The nurse wants something more than the irreducible minimum of reward, and hence the necessity for an inquiry by persons free from monetary shackles. Such an inquiry it is within the power of the College to inaugurate, and, from what I am able to glean at this early stage, is in contemplation.

### Two Important Factors.

At this juncture, and before any definite steps are taken, may I be permitted to offer one or two suggestions for the consideration of all concerned? I have yet no idea as to what form the proposed committee will take, or of what persons, or even of what sort of persons, it will consist. But, obviously, its constitution is a matter of supreme importance, the only equally important factor in the case is the strength of the College Register. The war has taught us the necessity for large armies and likewise for efficient generalship. It is for the nurses to provide the recruits and for the College to select the generals.

### Essentials of Inquiry Committee.

The essential qualifications of an effectual Committee of Inquiry, in my opinion, are (i) that it should be numerically small; (ii) that it should consist of persons fully alive to the potentialities of the nursing profession in connection with future schemes of reconstruction; (iii) that it should include in its membership at least one woman of outstanding ability, in close touch with women's movements at home and abroad. I shall probably incur severe hostile criticism when I further suggest that it is not essential, if it is even desirable, that hospital matrons or nurses should be included in any economic inquiry committee that may be formed. The science of economics and the science of nursing are quite apart and unrelated. Nurses are not business women, and the atmosphere that surrounds the zealous matron corresponds more closely with the cloister than with the mart. All that the Committee of Inquiry need to realise is that the nation requires a high standard of recruits in its nursing world—women on whom municipalities may depend in carrying out ambitious and thorough schemes of reconstruction and reform after the war. Having realised this they will be in a position to make such recommendations as will, if adopted, ensure the employment of the best, not of the second best, and certainly not of women who have turned out failures in their early endeavour, and who turn to nursing in the last resort, at the age of three or four and twenty, as something to fall back upon as a career.

### Capacity of the Witnesses.

So much for the committee. The nurses can fulfil their part best in the capacity of witnesses. Evidence ought to be heard not only from every part of the kingdom, but from every part of the Empire. Witnesses should be invited from the United States of America, so that when the committee makes its report the whole subject may be under review. The selection of suitable witnesses will be a difficult and delicate task, especially within the kingdom, having in view the present unorganised state of the profession. The College of Nursing is heartily to be commended for undertaking so great a task at this early stage in its history, and British nurses owe to it their loyalty and their gratitude, the expression of which can only be conveyed by promptly and in crowds rallying to its support. The more powerful the College the more authoritative will be its recommendations to the nation. Despite the fact that so far only twelve thousand nurses have allied themselves with the College, I suggest that witnesses be summoned or invited to attend on a thoroughly democratic basis. Let the matrons speak on their own behalf for matrons, sisters for sisters, and both on behalf of probationers, and let witnesses be chosen by popular election.

### Ierne in Modest Mood.

These passing thoughts, with the utmost modesty, I submit for consideration. Now is the time for articulation, for suggestion, for criticism. I am neither bigoted nor biassed; and I assure my readers that none more than I shall welcome criticism. I confess to having learnt more valuable lessons from hostile criticism in my life than from any other source.

